# Substance Use among Emerging Adults during the COVID-19 Pandemic: A Review through the Lens of Sustainable Development Goals

**DOI:** 10.3390/ijerph20196834

**Published:** 2023-09-26

**Authors:** Ajith K. Remesan, Varalakshmi Chandra Sekaran, Teddy Andrews Jaihind Jothikaran, Lena Ashok

**Affiliations:** 1 Department of Health Policy, Prasanna School of Public Health, Manipal Academy of Higher Education, Manipal 576104, India; ajith.remesan@learner.manipal.edu; 2 Department of Social and Health Innovation, Prasanna School of Public Health, Manipal Academy of Higher Education, Manipal 576104, India; teddy.andrews@manipal.edu (T.A.J.J.); lena.ashok@manipal.edu (L.A.)

**Keywords:** substance use, mental health, sustainable development goals, emerging adults, COVID-19

## Abstract

Public health initiatives, including lockdowns to reduce the spread of COVID-19, have resulted in societal stressors like loneliness, job loss, and economic collapse linked to worsening mental health outcomes, such as depression, anxiety, and coping-related substance use. The various psychosocial stressors caused by the pandemic have potentially led to the increased use of substances across the globe, particularly among emerging adults. The current study summarises the literature on substance use among emerging adults during the COVID-19 pandemic. The terms “coronavirus”, “COVID-19”, “substance use”, “substance abuse”, “drug use”, drug abuse”, “emerging adults”, and “young adults” were all used in different combinations throughout the search, using the Scopus, PubMed, and JSTOR databases. Using this method, a total of 28 English-language citations published between 2020 and 2023 were obtained. Following a study of the articles mentioned above, 16 papers were removed. The remaining 12 papers were included in this review. Even though substance use dropped when COVID-19 lockdowns began, it rose when restrictions were removed, particularly among those with prior substance use. Studies related to mental health need to assess substance use, as many emerging adults use substances to cope with distress, including isolation and loneliness, which are part of the current mental health crisis among emerging adults.

## 1. Introduction

The World Health Organization (WHO) declared COVID-19 a worldwide pandemic on 11 March 2020 [1]. The pandemic had a long-term impact, which affected all aspects of human life and prevented all developmental ventures, including the Sustainable Development Goals (SDGs). The global community established the SDGs in 2015 to enhance the quality of daily life for all citizens and to continue on the unfinished agenda of the Millennium Development Goals (MDGs), in which SDG 3 primarily aims at promoting well-being and ensuring healthy lives [2]. SDG 3 prioritised treating and preventing substance use; however, contending with the COVID-19 pandemic was the primary global priority during the outbreak. As a result, meeting the SDGs by the deadline of 2030 has become secondary [3]. Public health initiatives, including lockdowns to reduce the spread of the illness, had resulted in societal stressors like loneliness, job loss, and economic collapse linked to worsening mental health outcomes, such as depression, anxiety, and coping-related substance use [4].

The various psychosocial stressors caused by the pandemic have potentially led to the increased use of substances across the globe. The Centre for Disease Control and Prevention (CDC) estimated that 13% of Americans had started or increased their substance use to deal with the stress imposed by the pandemic [5]. According to the Substance Abuse and Mental Health Services Administration’s (SAMHSA) study, 15.3 million emerging adults (45.8%) had a substance use disorder or co-occurring mental illness in 2021 [6]. Substance use among emerging adults (i.e., age 18–25 years) was a growing concern marked by high rates of alcohol and tobacco use, which are the most accessible substances. Emerging adulthood refers to the age group between 18–25 years. Even though substance use appears to be initiated at late adolescence, it appears to peak during emerging adulthood and after [7,8,9]. In the Indian context, there was an increase of 28% in substance use among emerging adults. The number of people who sought treatment for substance use during the pandemic also increased [10]. Research evidence also showed that emerging adults had higher rates of psychological distress related to the pandemic in comparison with other age groups, which aligns with other studies on pandemics and natural calamities in the past [11,12,13]. Given the widespread increase in substance use among emerging adults since the outbreak, this trend demands further attention [14,15].

Nevertheless, on one side, studies showed a significant increases in substance use among emerging adults to deal with pandemic-related stressors [16,17]; however, on the other side, different coping mechanisms were also utilised broadly by emerging adults [18]. These observations provide an inconclusive picture of the extent to which the pandemic has led to an increase in substance use and whether the use of different substances acted as a coping mechanism among emerging adults during the pandemic. Considering these critical research findings, the present review aims to answer the questions surrounding the substance use trends and coping strategies among emerging adults during the COVID-19 pandemic with regard to SDG 3.

## 2. Materials and Methods

### 2.1. Search Methodology and Article Selection

Concerning the COVID-19 pandemic, the current study summarises the literature on substance use among emerging adults. The following search string was used across databases Scopus, PubMed, and JSTOR: ((coronavirus) OR (COVID-19)) AND ((substance use) OR (substance abuse) OR (drug use) OR (drug abuse)) AND ((emerging adults) OR (young adults)). Using this method, a total of 28 English-language citations published between 2020 and 2023 were shortlisted. Following a review of the articles mentioned above, 16 papers were removed, including 5 review articles, 1 letter to editor, and other articles which addressed aspects of COVID-19 which were not pertinent to this review, such as managing COVID-19 treatment, online education, and working from home. The remaining 10 publications, except review papers ,were thoroughly examined but were not found to be pertinent to emerging adults’ substance use during the pandemic.

### 2.2. Methodological and Thematic Analysis of Selected Articles

**Figure 1:**
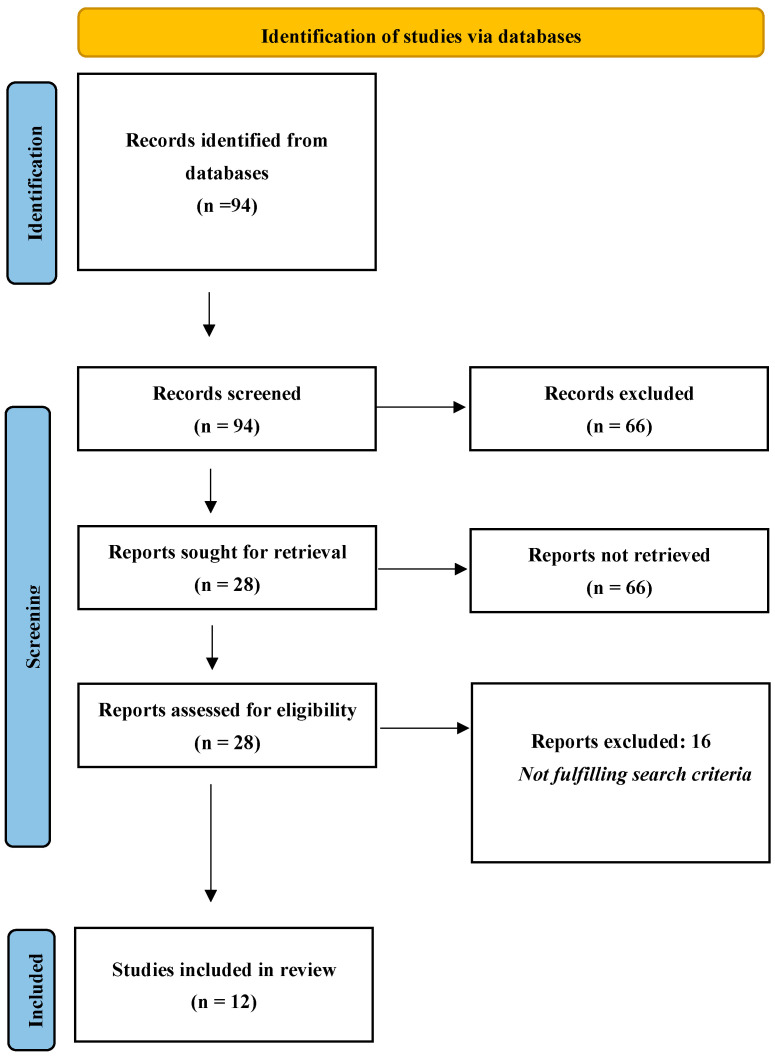
PRISMA flow diagram: The flow of information across various stages of the review [19].

In total, 12 papers were included in this review (Figure 1). In terms of the methodology employed in these studies, 8 were cross-sectional studies and 3 were longitudinal cohort studies, including a mixed methods study and 1 empirical study. Most papers were from the USA (seven cross-sectional studies and two longitudinal cohort studies). There was one publication from India and two from Canada.

### 2.3. Inclusion and Exclusion Criteria

We focused on studies reporting data on substance use among emerging adults during the COVID-19 pandemic. The review included studies incorporating the age group 18–25 years (any gender). We excluded studies if they failed to report findings relating to substance use among emerging adults during COVID-19 or were not published in English. We excluded the studies needing more information to judge the eligibility criteria from our review.

## 3. Results and Discussion

The selected papers were divided into three themes according to the areas they discussed. They are the trends and reasons for the use of substances among emerging adults during COVID-19, substance use and psychological distress during COVID-19, and COVID-19 and coping mechanisms among emerging adults. Along with these themes, the review also tries to highlight how COVID-19 affected the Sustainable Development Goals in association with substance use.

### 3.1. Trends of Substance Use among Emerging Adults Due to COVID-19 Restrictions

The commencement of COVID-19 significantly impacted the use of substances. Among those who had previously used substances, there was a 23% rise in alcohol use and a 16% increase in other substance use, including tobacco, even though cannabis usage did not vary when the lockdowns were imposed [20]. There was a 25% increase in vaping among emerging adults during the pandemic. Most of them believed that vaping would not cause any health issues compared with smoking tobacco [21]. Even though the number of emerging adults who initiated substance use during the pandemic is low compared with existing users, the COVID-19-related concern and anxiety were the highest among people who began using substances during the COVID-19 pandemic compared with those who had previously used substances and those who had never used them [4,21]. This might show a pattern in use before and during the pandemic. However, it may also indicate a zero net impact of higher demand and decreased supply of cannabis during lockdown, since borders were closed, distributors had a more difficult time travelling, and prices increased because of increasing demand [22,23]. People who isolated themselves consumed 26% more alcohol than they would generally consume to cope [24]. As alcohol and tobacco were the major substances available during the restriction, the consumption of these substances during COVID-19 increased substantially among emerging adults. Among people aged 15 to 34 years, emerging adults reported increased use of substances such as tobacco and alcohol compared with those aged 55 years and older [25]. According to excised department records from March 2020 to April 2020, the amount of wash confiscated (the foundation for producing locally made alcohol-arrack in India) progressively climbed from 160 to 6000 litres per day [26]. Emerging adults who used alcohol prior to COVID-19 reported the greatest increase in their usage of alcohol during the pandemic. They also reported a decline in the usage of inhaled substances, such as e-cigarettes [27]. In India, two-thirds (67%) of those who smoked tobacco were unaware that tobacco use could put them in the higher-risk groups against COVID-19. Only 30% of consumers believed that the restrictions during the pandemic had influenced their tobacco usage, with the most prevalent consequences being a lack of availability of tobacco products and higher pricing. While some users saw this as a chance to stop their usage, others reported increased tobacco usage due to stress during the pandemic [28]. The emerging adults living with parents were at risk of substance use if any of the parents were using substances or if the emerging adult was under the pressure of pandemic results such as isolation. Substance use also raised the risk of infectious illnesses such as HIV/AIDS, tuberculosis (TB), and COVID-19, as well as noncommunicable illnesses [29]. Emerging adults who provided specific explanations for their increased substance use cited boredom, spending more time at residence, lacking other activities, isolation, and experienced significant disruptions to their daily life as essential drivers [13]. Factors such as depressive symptoms, perceived stress, stress management, internet use, and lack of peer support during the COVID-19 pandemic also significantly increased substance use among emerging adults [5,29].

To prevent a surge in substance use leading to public health concerns, various groups, including local and state governments, healthcare professionals, and law enforcement, need to work together to mitigate the effects of stressors caused by future pandemics. The pandemic’s acute health consequences provide an apparent response to coordinating our political and societal actions to prevent additional damage. Being ready for undesirable events allows us to be prepared for adverse outcomes.

### 3.2. Substance Use and Psychological Distress during COVID-19

Secondly, much of the literature spoke about the psychological impact caused by COVID-19. The pandemic has had severe repercussions all over the world, and there was uncertainty regarding the spread of the illness and the pandemic’s impact on society. These constraints compounded the numerous challenges that have troubled society for years, such as inadequate access to health care, more comprehensive socioeconomic division, and rising rates of depression and anxiety owing to a lack of social contact [5]. The emergency restrictions, including lockdowns, resulted in unemployment, economic fallout, indebtedness, homelessness, and poverty, which also led to different kinds of psychological distress and substance use [30].

There were various reasons for the respondents’ substance use and psychological distress during the outbreak. The emotional stress brought on by worries about how the pandemic would directly impact affected individuals and loved ones, the disease’s political and social effects, stress related to connectedness, lack of social events, interpersonal pressure, and changes in routine and sense of normalcy were a few among them. It was also noted that substance use was a major reason for increased psychological distress in society and vice versa [13,31]. Those who were having higher anxiety and depression used more alcohol, and those who had social support used less substances during COVID-19 [16]. As the restrictions were implemented to reduce the spread of disease, social connectedness was also controlled, which may have affected emerging adults in the form of social support. Emerging adults who initiated substance use during pandemic as a coping mechanism and those who were accessing care at substance use treatment centres were particularly at risk during the pandemic [4]. The mental health of substance users was severely impacted by the change in substance availability and scarcity during the initial phase of restrictions, perhaps leading to suicidal thoughts and self-harm behaviours. As reported by R. Sharma et al. (2021), there has been a 50% increase in patients seeking treatment in the emergency rooms of hospitals for problems related to alcohol and substance withdrawal. Studies in India also revealed a similar sharp increase in substance use cases and registrations for treatment at deaddiction facilities following the pandemic [10,31].

Research on the psychological consequences suffered by infected individuals during previous pandemics was consistent with the increases in depression symptoms and perceived stress throughout COVID-19 [18]. Symptoms of anxiety disorder or depressive illness, substance use initiation or increase to cope with COVID-19-related stressors, and profound suicidal impulses were most often reported by those aged 18–24 years, and the prevalence declined gradually with age [32]. The majority of studies on the possible influence of the outbreak on the number of suicides have been carried out in high-income nations. According to the findings, the rate of suicide was not increased during the first few months of the outbreak, with Hungary and Japan being significant outliers [33,34]. In fact, despite initial concerns, there was a drop in the rates of suicides in several regions, and potential explanations for this drop in other places in the world consist of timely responses by authorities to the pandemic’s social, economic, and psychological impacts, such as improved mental health services, the commencement of financial assistance, and a sense of solidarity in confronting the pandemic’s common challenges [35]. The data from India seems to stand apart from other parts of the world, as the number of suicides in India increased throughout the pandemic, and males were far more likely to commit suicide than females. Male suicides in India can be linked to the traditional position of males as breadwinners of the family and the stressors of taking care of the family. While untested, the economic effects of the pandemic, such as job loss and associated role pressure and guilt, may have been felt more intensely by men [36].

Over the last decade, India’s mental health services have grown, and new efforts were launched during the COVID-19 pandemic. However, a lack of mental health specialists and other health system infrastructure shortages continues in most parts of the country, as in many other middle-income countries. Compared with settings with better-resourced mental health systems, the mental health system in underdeveloped sections of India was not as ready to respond to the rising psychological burden in society during the COVID-19 pandemic, which may have led to an increase in suicide rates. Furthermore, mental health concerns are severely stigmatised in India, contributing to a lack of help-seeking and care provision.

### 3.3. COVID-19 and Coping Mechanisms among Emerging Adults

The third theme that emerged from the review was about the coping mechanisms that emerging adults adopted to adjust to the impact of the pandemic.

Emerging adults used different coping mechanisms during the pandemic to cope with psychological distress, which could be adaptive or maladaptive coping strategies. Substance use seems to be one of the major maladaptive coping mechanisms that were adopted by emerging adults. Along with that, self-blame, behavioural disengagement, and denial were also connected to the maladaptive coping mechanisms, which were mainly induced by substance use [37]. Although there were no statistically significant differences in adaptive coping across groups, emerging adult men appeared to engage in more maladaptive coping. This finding can be explained by the fact that emerging adult women appeared to have dealt more positively with the perceived COVID-19-associated risks than males during this pandemic and were more involved in developing coping mechanism methods than males. However, this was not the case for every emerging adult; there were emerging adults who adopted adaptive coping strategies as well [38]. Examples of adaptive coping include self-distraction, emotional support, acceptance, and religion. During the initial COVID-19 restriction phase, despite experiencing significantly greater anxiety symptoms than emerging adult men, emerging adult women (66%) were more likely to engage in emotion-focused coping techniques such as expressing emotions and meditation. Men, on the other hand, adopted problem-focused solutions, such as challenging the problem. However, most problem-focused coping strategies were associated with higher anxiety levels in both men (34%) and women (66%). This might be because it was challenging to organise and ask for practical assistance from others during the lockdown periods when people were forced to remain socially isolated [39]. These findings revealed a link between COVID-19-related anxiety, substance use, and behavioural disengagement, which also implies that when people adopt behavioural disengagement to deal with the COVID-19 threat, they put themselves in the risk categories. This is consistent with prior studies, which identified behavioural disengagement as a maladaptive coping mechanism that indicates a tendency to reduce one’s coping efforts, leading to the individual giving up. According to Wardell, when people experience high anxiety levels due to a pandemic, they are more prone to use substances to relieve their worries.

Moreover, stress and anxiety among emerging adults appeared to have linked with increased internet use [40]. Use of the Internet acted as both adaptive and maladaptive coping mechanisms. During the pandemic’s restrictions, individuals had little influence over their own lives and faced significant educational stress and concern; greater use of the Internet might have been an easy coping strategy. Controlling their Internet activities helped them to deal with their helplessness throughout the pandemic [37]. Furthermore, while virtual achievement through gaming and blogging may have been used to cope with low self-esteem and life satisfaction in the actual world, it always carried the danger of problematic Internet usage and substance use. Reports suggest a positive correlation between Internet use, alcohol use, and smoking. During the pandemic, 25% of emerging adult substance users switched to vaping, as most of them used different online platforms to order e-cigarettes [21]. The lack of availability and access to other substances due to the restrictions could be the reason for this change. Due to the reduced face-to-face contact, participation in other digital endeavours such as using social media, playing video games, and online education was the primary resource for social connectedness among emerging adults. This increased problematic Internet-related coping behaviour and substance use during the COVID-19 pandemic, placing emerging adults at a higher risk of Internet addiction and substance use [41].

Most emerging adults are unfamiliar with adaptive coping techniques and tend to follow maladaptive coping. An increase in global investment must back the effort to promote better outcomes. Unfortunately, this issue highlights an ongoing worldwide absence of mental health services. According to the World Health Organization’s most current Mental Health Atlas, governments worldwide spent just over 2% of their health budgets on mental health in 2020, and several low-income nations were reported to have less than one mental health practitioner per 100,000 people.

### 3.4. COVID-19 and Substance Use: Challenges to Achieve Sustainable Development Goals (SDGs)

Even before the pandemic, substance use was always one of the most significant risk factors for the health of populations globally. The world is still reeling from many challenges to humankind after the pandemic. COVID-19 has slowed down the journey to achieving Sustainable Development Goals by 2030, which is a vital crossroads in our development phase, and one of the major reason for this was the increased substance use among emerging adults [42]. More individuals are taking substances, and more illicit substances are available than ever. Emerging adulthood and young adulthood are two age groups with potentially differing levels of vulnerability and environmental impact, and they also comprise an overall population comprising a group where the use of substances such as tobacco and alcohol is deemed illegal and another where it is controlled. The legal age for using substances like alcohol and tobacco varies by country.

The increase in substance use during the pandemic affected achieving almost all the SDGs. Substance use raised the risk of infectious illnesses such as HIV/AIDS, tuberculosis (TB), and COVID-19, as well as noncommunicable illnesses such as cardiovascular disease, cancer, complications of liver disease, pancreatitis, and diabetes. It was also associated with additional hazards, such as lack of physical activity and poor diet. It impaired brain function and contributed to impaired driving, raising the chance of getting involved in an accident with a blood alcohol content of more than zero. Alcohol-related harm imposes a substantial financial cost on healthcare systems [29]. Globally, at least 283 million persons aged 15 and above suffer from an alcohol consumption problem, accounting for 5.1% of all adults. Alcohol intake causes mortality and disability at a young age. Substance use accounts for roughly 13.5% of all fatalities in adults aged 20 to 39 years [43].

The COVID-19 outbreak exposed human fragility, imposing pressure on health institutions and stretching social safety nets to the breaking point. The pandemic’s economic crisis also played a vital role in boosting individuals’ substance usage and motivated emerging adults to participate in more drug trafficking and related criminal activity. Amid the worst socioeconomic crisis in centuries, governments cannot afford to overlook the hazards of illegal, illicit substances to public health and safety. Poverty, restricted access to higher education and career prospects, stigma, and social exclusion contributed to the hazards and impacts of substance use worldwide, widening disparities and moving us farther from achieving the SDGs [44]. Based on information gathered by the Centre for Monitoring Indian Economy (CMIE), the connection between unemployment and substance use cannot be neglected, as India’s unemployment rate jumped to 7.8% in October 2022 from 6.4% the previous month. While the urban unemployment rate fell from 7.7% to 7.2%, it increased from 5.8% to 8.0% in rural areas of India. Also, there was an increase in registered crimes, such as sexual assault, abduction, crimes against minors, and murder in 2021, with the actual figure thought to be substantially higher, because many crimes go undetected; this could be due to the increase in substance use, as the literature mentions a correlation between unemployment and substance use [45].

Substance use continues to be a severe challenge to achieving the SDGs. So far, governmental responses have been insufficient in safeguarding people from the dangers of substance use. The WHO’s initiatives, including the “Global Alcohol Action Plan” established in 2022 and incorporating the most affordable alcohol control measures focused on lowering population-level alcohol consumption, will aid in achieving the SDGs by 2030. Alcohol policy is a driver of long-term growth and equity (Table 1).

## 4. Conclusions

As this review highlights, substance use and psychological distress increased during COVID-19 among emerging adults. Though there was a decrease in substance use during the initial phase of the COVID-19-related pandemic restrictions, it increased sharply once the restrictions were removed, particularly among those who had prior use of substances. This shows that restricted access was key in reducing usage. Concomitantly, there was an increase in psychological distress noted during the pandemic, which was augmented by several other pandemic-related impositions. During the pandemic, considerable modifications and adaptations where required in everyone’s life, and this review demonstrates how those changes negatively affected certain people more than others. The tendency to utilise more maladaptive coping mechanisms than adaptive coping ones needs to be addressed in the future. This review also shows that the influence of the pandemic on an individuals’ psychological distress and coping methods varied by age and gender, and the elder population experienced lower psychologically detrimental issues during the new normal era than emerging adults. Of note is the finding that emerging adult women reported using more emotion-focused coping to adapt to the pandemic restrictions and psychological distress than emerging adult men, who used more problem-focused coping mechanisms. Emerging adult men who utilised the Internet frequently had higher maladaptive coping. As a result, the analysis gives additional paths for understanding psychological distress and coping in various segments of the community. Based on these findings, personalised interventions concentrating on coping techniques may be created and implemented for the affected population. More studies on the elements which make these people vulnerable can be undertaken.

Moreover, preventive technics for managing future crises may be taken. It is also important to initiate discussions on new or existing policy ideas addressing health inequalities, such as expanding resources available for diagnosis and treatment choices for populations at elevated risk for psychological distress and maladaptive coping during pandemics, which may be a threat in the future. Resources such as social support, extensive treatment choices, telemedicine, and harm reduction programmes are crucial and should continue to be available to reduce the possible adverse effects of excessive substance use connected to pandemics. Improving emerging adult mental health needs to be a key priority in the health sector. We must increase our understanding of how effectively we can serve individuals experiencing psychological distress. Data from the review emphasise the necessity of furthering our knowledge of how substance use varies during pandemics and what infrastructure may be established to identify and help people with mental health difficulties.

To achieve the SDGs’ transformational goal by 2030, most nations’ priorities must be significantly realigned towards long-term, cooperative, and substantially accelerated action. As noted by UN Secretary-General Antonio Guterres, “Everything we do during and after this crisis must be with a strong focus on building more equal, inclusive and sustainable economies and societies that are more resilient in the face of pandemics, climate change, and the many other global challenges we face” [46].

## 5. Limitations

The interpretations offered here should be considered within the context of some limitations. Studies meeting the inclusion criteria were sourced from different countries but did not cover the whole of the globe. We also could not focus on a particular geographical area due to a lack of data available. Thus far, a few papers have discussed substance use and Sustainable Development Goals (SDGs) in the post-COVID-19 era. We could only evaluate articles available in English at the time of the search, which may have omitted studies. Most of the studies included relied on self-report in response to questionnaires to assess substance use and other associated concerns. It is commonly known that when asked about their substance usage, people frequently underreport it due to social desirability biases.

## Figures and Tables

**Table 1:** Summary of the articles selected for the review.

Author	Country of Origin	Population(s)	Study Methodology	Major Study Instruments	Results
Khauli et al., 2023 [22]	USA	Age 18–25 years N = 849	- Online survey - Cross-sectional	(1) DDQ—Daily Drinking Questionnaire. (2) AUDIT—Alcohol Use Disorders Identification Test.	- Young adults at risk for AUD prior to the arrival of COVID-19 experienced greater increases in drinking. - Increase in drinking were heterogeneous, impacted by different roles and environmental factors.
E. Greenglass et al., 2022 [40]	Canada	Age 18 and above N = 730	- Online Survey - Empirical Study	(1) Avoidance Behaviour Scale.	- COVID-19 was associated with anxiety and maladaptive coping, such as substance use and behavioural disengagement. - To the extent that individuals manage their anxiety through substance use or behavioural disengagement, they were less likely to avoid situations that increased their risk of infection.
K. Romm et al, 2022 [1]	USA	Age 18–34 years N = 1082	- Online survey - Cross-sectional	(1) PHQ—The Patient Health Questionnaire. (2) ACE—Adverse Childhood Experiences.	- There was an increase in alcohol use and decrease in vaping products usage. - Depressive symptoms predicted increases in alcohol use.
M. Sylvestre et al., 2022 [25]	Canada	Age 12–34 Years N = 1294	- Longitudinal Cohort Study	(1) NDIT—Nicotine Dependence in Teens.	- Use of cannabis and e-cigarettes increased by 5.6% and 1.6%, respectively. - Low education and living alone were associated with higher risks of initiated/ increased use of most substances.
Horigian et al., 2021 [32]	USA	Age 18–35 years N = 1008	- Online Survey - Cross-sectional	(1) UCLA Loneliness Scale (version 3). (2) AUDIT—Alcohol Use Disorders Identification Test.	- Feelings of loneliness, substance use, anxiety, and depression increased, while experiencing a decrease in feelings of connectedness. - Change in loneliness was associated with changes in alcohol use.
K. Romm et al., 2021 [30]	USA	Age 18–34 Years N = 1082	- Longitudinal Cohort Study		- Among the respondents, 41.3% increased alcohol use, 47.2% decreased physical activity, 74.0% were more sedentary, and 34.7% experienced poorer nutrition, all of which predicted by greater depressive symptoms.
L. Papp et al., 2021 [38]	USA	Age 18–21 Years N = 295	- Online Survey - Cross-sectional	(1) DAST-10—Drug Abuse Screening Test	- The increase in substance use was particularly elevated among young adults who reported higher levels of loneliness and worry about their health. - Alcohol and marijuana are among the most commonly used substances by college students.
N. Charles et al., 2021 [18]	USA	Mean Age 21.16 Years N = 774	- Online Survey - Cross-sectional	(1) CCSM—Cross-Cutting Symptoms Measure. (2) AUDIT—Alcohol Use Disorders Identification Test. (3) PSS—Perceived Stress Scale.	- Use of alcohol and anger rate was high among college students. - Along with alcohol misuse, higher levels of depression and perceived stress were also reported.
R. Emery et al., 2021 [13]	USA	Age 21–29 Years N = 670	- Longitudinal Cohort Study - Mixed-Methods Study	(1) COVID-19—EAT Survey	- 84% reported that the pandemic influenced their mood or stress. - Majority of participants reported their motivations for substance use were boredom, more time, and having nothing else to do.
S. Singh et al., 2021 [37]	India	Age 13–26 years N = 1027	- Online Survey - Cross-sectional	(1) PSS—Perceived Stress Scale (2) Brief COPE—Coping Orientation to Problems Experienced	- Emerging adult males had higher maladaptive strategies. - Substance use was moderately positively correlated with maladaptive coping.
P. Sharma et al., 2020 [27]	USA	Age 18–25 years N = 1018	- Online Survey - Cross-sectional	(1) Self-developed Survey Tool (2) UCLA—Loneliness Scale	- Largest reporting change was in alcohol use - Young adults often indulge in drinking behaviour to cope with aversive mood states, which prolongs or worsens negative affective states and further drinking.
W. Lechner et al., 2020 [16]	USA	Age 18–25 Years N = 1958	- Online Survey - Cross-sectional	(1) TLFB—The Timeline Follow-Back Interview (2) PHQ-9—The Patient Health Questionnaire-9.	- An increase in alcohol consumption was observed following the announcement of campuses closing. - Students with higher levels of depression and anxiety reported greater increases in alcohol use over time than those with lower levels of distress.

## Data Availability

Not applicable.
